# Influence of insurance type on rate and type of initial concussion-related medical visits among youth

**DOI:** 10.1186/s12889-021-11586-y

**Published:** 2021-08-18

**Authors:** Chris Radlicz, Kenneth Jackson, Amanda Hautmann, Junxin Shi, Jingzhen Yang

**Affiliations:** 1 Center for Injury Research and Policy, Nationwide Children’s Hospital, 700 Children’s Drive, RBIII-WB5403, Columbus, OH 43205 USA; 2 Biostatistics Resource, Nationwide Children’s Hospital, Columbus, OH USA; 3 Center for Biostatistics, The Ohio State University, Columbus, OH USA; 4 Department of Pediatrics, College of Medicine, The Ohio State University, Columbus, OH USA

**Keywords:** Traumatic brain injury, Insurance, Health care utilization, Children

## Abstract

**Background:**

A growing number of studies report increased concussion-related health care utilization in recent years, but factors impacting care-seeking behaviors among youth following a concussion are not well described. This study aimed to evaluate the influence of insurance type on the rate and type of initial concussion visits and the time from injury to the initial visit in youth.

**Methods:**

We extracted and analyzed initial concussion-related medical visits for youth ages 10 to 17 from electronic health records. Patients must have visited Nationwide Children’s Hospital’s (NCH) concussion clinic at least once between 7/1/2012 and 12/31/2017. We evaluated the trends and patterns of initial concussion visits across the study period using regression analyses.

**Results:**

Of 4955 unique concussion visits included, 60.1% were males, 80.5% were white, and 69.5% were paid by private insurance. Patients’ average age was 13.9 years (SD = 3.7). The rate of the initial concussion visits per 10,000 NCH visits was consistently higher in privately insured than publicly insured youth throughout the study period (*P* < .0001). Privately insured youth had greater odds of initial concussion visits to sports medicine clinics (AOR = 1.45, 95% CI = 1.20, 1.76) but lower odds of initial concussion visits to the ED/urgent care (AOR = 0.74, 95% CI = 0.60, 0.90) than publicly insured youth. Days from injury to initial concussion visit significantly decreased among both insurance types throughout the study (*P* < .0001), with a greater decrease observed in publicly insured than privately insured youth (*P* = .011).

**Conclusions:**

Results on the differences in the rate, type, and time of initial concussion-related visits may help inform more efficient care of concussion among youth with different types of insurance.

## Background

Concussion, a form of mild traumatic brain injury, affects approximately 2 million youth in the United States annually [1, 2]. In recent years, youth concussion has received increased attention due to growing public awareness of concussion-associated short- and long-term effects on physical, cognitive, emotional, and sleep health [3, 4]. If left untreated or mismanaged, a concussion can impair quality of life beyond acute symptomology [4–6] and increase the risk of repeat injury [7]. To help mitigate the consequences of concussion, all 50 states and the District of Columbia enacted concussion laws between 2009 and 2014 to promote secondary prevention efforts [8]. These laws share three core elements: (1) removal from play following a suspected or apparent concussion; (2) medical clearance approved by a licensed health professional to return to play; and (3) education for parents, student-athletes, and coaches regarding concussion signs and symptoms [8]. Early evaluation of these laws show an increase in youth concussion rates and related healthcare utilization attributed to increased recognition, awareness, and reporting [9–15]. Though a growing number of studies report increased concussion-related healthcare utilization, how these care-seeking behaviors are influenced by insurance status and type remains undetermined [10, 12, 16].

Existing evidence indicates that publicly insured youth seeking care for concussion may have limited provider options, decreased outpatient rehabilitation choices, increased barriers to specialty care, and increased wait times [17–20]. Furthermore, publicly insured youth may have higher unmet needs and poorer outcomes after concussion compared to their privately insured counterparts [21]. Concussion-related visits for both publicly and privately insured youth increased in recent years due, in part, to concussion laws requiring youth to seek medical care for concussive injury in order to return to play [10, 15, 22]. Moreover, studies indicate an increase in the initial concussion-related emergency department (ED) visits for publicly insured youth relative to privately insured youth [15, 22]. Publicly insured youth are more likely to use the ED as an initial point of entry for concussion treatment compared to privately insured youth, [23] while studies of privately insured youth show greater increases in primary care and specialty care [10, 24].

Though the number of youth concussion studies continues to rise, there remains a paucity of studies outside of the ED setting or in populations other than high school and college athletes [10, 14, 23, 24]. Moreover, the few studies analyzing the influence of insurance type on concussion-related care utilization forgo an examination of how insurance type guides concussion care seeking behaviors, where care is sought, and when care is sought [14, 23]. Using the electronic health record (EHR) system in a large pediatric healthcare network, we aimed to describe the influence of insurance type on the rate of the initial concussion visit, the type of initial visit sought, and the time from injury to the initial concussion visit. We hypothesized that a higher rate of initial concussion visits would be observed among privately insured youth than publicly insured youth; that compared to privately insured youth, publicly insured youth would be more likely to visit the ED or urgent care but less likely to visit sports medicine clinics for their initial concussion care; and that days from injury to initial concussion visit would decrease throughout the study regardless of insurance type.

## Methods

### Study data and case definition

We retrospectively analyzed medical visits for pediatric concussions extracted from electronic health records (EHR) collected as part of routine clinical care for patients seen at Nationwide Children’s Hospital (NCH). NCH EHR uses a comprehensive and integrated set of clinical software systems to manage and record various patient care data domains such as demographics, medical visits, diagnoses, orders, and provider information that can be extracted to support data-driven research endeavors. For this study, we defined medical visits for concussions using the following International Classification of Diseases, Ninth and Tenth Revisions, Clinical Modification (ICD-9-CM and ICD-10-CM) codes: 850.0, 850.1, 850.11, 850.12, 850.2, 850.3, 850.4, 850.5, 850.9, and those beginning with S06.0 [10]. We extracted the following data elements from the EHR for each concussion: basic demographics (e.g., date of birth, sex, race) and medical visit information (e.g., diagnostic codes, date of injury, date and type of visit, date of first visit, date of symptom resolution, and payor). Additional data regarding the total number of all-cause first medical visits by insurance type during the study period were also extracted. This study received the approval for a w*aiver* of informed *consent* (IRB18–01171) from the Institutional Review Board of the primary authors’ institution.

### Study population

The population in this study was youth ages 10 to 17 years old who had a confirmed isolated concussion diagnosis, had medical visits for one or more concussions between July 1, 2012, and December 31, 2017 (not necessarily date of injury), and visited one of seven NCH concussion clinics at least once for their concussion treatment. Each medical visit for a unique concussion was determined by the date and time of the visit. Patients with repeated concussions were preceded by at least 90 days without an additional concussion diagnosis. We used the date of injury, date of the first visit, and date of symptom resolution to differentiate each unique concussion and included these in the analysis.

Patients were excluded if: 1) they were also diagnosed with a more severe TBI within 2 weeks of the initial concussion visit; or 2) they were receiving ongoing concussion treatment during the study period, but the patient’s first medical visit occurred before July 1, 2012.

**Fig. 1:**
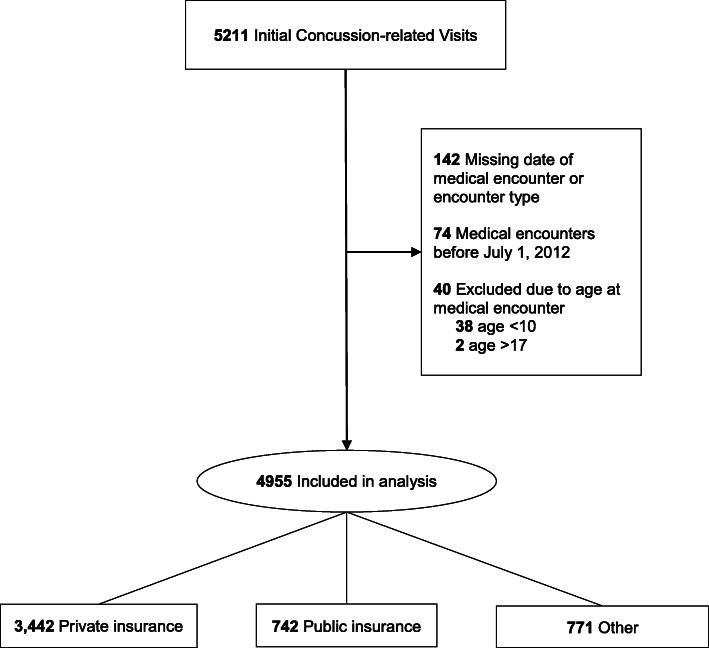
Flow Chart of Initial Concussion-related Visits for Inclusion Analysis

A total of 5211 initial concussion visits were identified, with 4955 meeting criteria for analysis. Figure 1 presents the exclusion process for initial visits to arrive at the final sample used in the analysis.

### Study variables and measures

***Rates of initial concussion visits*** were calculated as the number of initial medical visits for concussions among youth with private insurance (or public insurance) in a year divided by the total number of initial medical visits of NCH patients with private insurance (or public insurance) in the same year, then multiplied by 10,000.

***Type of initial concussion visit*** was classified as 1 = Sports Medicine; 2 = ED, including ED or urgent care centers; and 3 = Other, for all other specialties [23].

***Days from injury to initial concussion visit*** were measured as the number of days from date of injury to date of first medical visit.

***Insurance type*** was measured based on insurance plans across all medical visits associated with each injury (1 = Public (e.g., Medicaid), 2 = Private, 3 = Self-pay, and 4 = Other). Self-pay and other were later collapsed together. Injuries with differing insurance plans across medical visits were labeled as other.

***Other variables*** included ***patient demographics*** (e.g., age, sex, race), ***season/quarter*** (i.e., January through March, April through June, July through September, and October through December) and ***calendar year of medical visit*** (calendar year from 2012 to 2017).

### Statistical analysis

We described and compared demographic and injury characteristics of the study participants across different insurance types using chi-square tests. We examined trends across years and their potential interaction with insurance type via statistical models. Specifically, we employed Poisson regression to model the rate of initial healthcare visits for concussions versus initial visits for all causes, logistic regression to assess the proportion of concussion-related initial visits to sports medicine at NCH (or to ED) vs. otherwise, and linear regression (with log-transformed outcome) to determine the number of days from injury to initial visit to NCH. In all regression analyses listed above, we used hierarchical modeling to account for a patient with multiple concussions by nesting these injuries within a patient and assessed the interactions between year and insurance type. Further, we adjusted for study year and season, patient age, sex, and race (patient race was not included in the Poisson regression analysis since denominator information was not available at that level). Data were analyzed using SAS (version 9.4, SAS Institute Inc., Cary, NC), and a statistical significance level was set a priori for each test at α = 0.05.

## Results

Of 4955 unique concussions included, 69.5% of initial concussion visits were paid by private insurance and 15.0% were paid by public insurance (Table 1). The “other” insurance group (*n* = 771, 15.6%), including self-pay (*n* = 43) and pay by mixed public or private insurance and/or self-pay (*n* = 728), was not included in the further regression analyses. Concussion patients were primarily male (60.1%), White (80.5%), and had an average age of 13.9 (SD = 3.7) at initial concussion visit.

**Table 1:** Patient Demographics by Insurance Type, 2012–2017 (*N* = 4955)

Characteristic	Private Insurance (***n*** = 3442)	Public Insurance (***n*** = 742)	Other (***n*** = 771)	***P*** Value^**a**^
Sex, No. (%)				< 0.0001
Male	2004 (58.2)	514 (69.3)	462 (59.9)	
Female	1438 (41.8)	228 (30.7)	309 (40.1)	
Age, No. (%), y				< 0.05
10–13	1365 (39.7)	310 (41.8)	309 (40.1)	
14–17	2077 (60.3)	432 (58.2)	462 (59.9)	
Race, No. (%)				< 0.0001
White	3003 (87.3)	434 (58.5)	550 (71.3)	
Black	217 (6.3)	203 (27.4)	133 (17.3)	
Multiple	148 (4.2)	64 (8.6)	70 (9.1)	
Other	46 (1.3)	9 (1.2)	8 (1.0)	
Unknown	28 (0.8)	32 (4.3)	10 (1.3)	
Ethnicity, No. (%)				< 0.0001
Non-Hispanic	3306 (96.1)	675 (91.0)	713 (94.8)	
Hispanic	12 (0.4)	35 (4.7)	13 (1.7)	
Unknown	124 (3.6)	32 (4.3)	27 (3.5)	
Study Year, No. (%)				< 0.0001^c^
2012^b^	367 (10.7)	70 (9.4)	88 (11.4)	
2013	659 (19.1)	118 (15.9)	164 (21.3)	
2014	610 (17.7)	146 (19.7)	163 (21.1)	
2015	643 (18.7)	132 (17.8)	152 (19.7)	
2016	641 (18.6)	153 (20.6)	127 (16.5)	
2017	522 (15.2)	123 (16.6)	77 (10.0)	

^a^ Based on chi-square tests of the distribution between insurance types

^b^ Quarter 3 and 4 only

^C.^*P*-value was based on counts at the quarter level

### Rate of initial concussion visit by insurance type

**Fig. 2:**
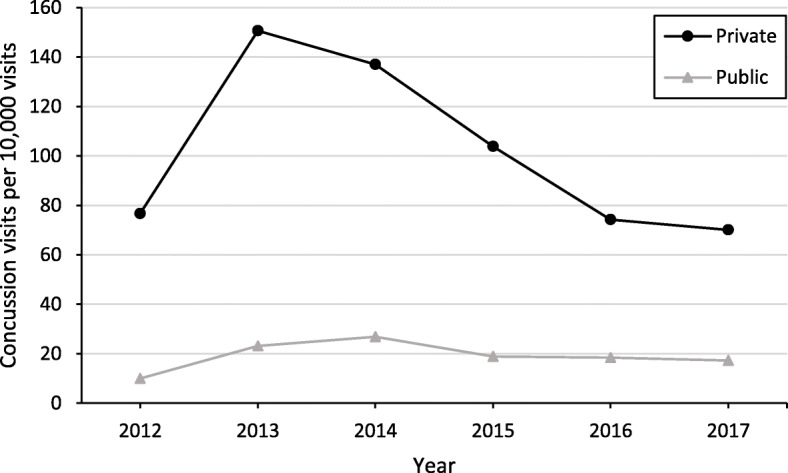
Rate of Initial Concussion-related Visit by Insurance Type, 2012–2017 (*N* = 4184)**.** Note: Rate of concussion-related visit was calculated as number of first visits among youth with concussions in a year divided by the total number of first visits of NCH patients in the same year, then multiplied by 10,000

The rate of the initial concussion visits per 10,000 NCH visits was consistently higher during the study period for privately insured youth compared to publicly insured youth (β = 1.9719, *P* < .0001) (Fig. 2). The overall rate of initial concussion visits decreased throughout the study period (β = − 0.0701, *P* < .0001), with a greater decrease in rate for privately insured visits compared to publicly insured visits (β = − 0.1008, *P* < .0001). Specifically, the rate of initial concussion visits among privately insured youth decreased from a peak of 150.7 per 10,000 in 2013 to 70.1 per 10,000 in 2017, while rate of initial concussion visits among public insurance decreased from 23.1 per 10,000 in 2013 to 17.2 per 10,000 in 2017.

### Type of initial concussion visit by insurance type

Privately insured youth were 1.45 times as likely to have initial concussion visits to sports medicine clinics (95% CI = 1.20, 1.76) but 0.74 times as likely to have initial concussion visits to the ED/urgent care (95% CI = 0.60, 0.90) as compared to publicly insured youth, after adjusting for patient age, sex, race, study year, and season (Table 2). Initial concussion visits to the ED and urgent care significantly increased from 2012 to 2017, with adjusted odds ratio (OR) of 1.19 (95% CI = 1.13, 1.25) for each advanced study year. However, initial concussion visits to sports medicine clinics significantly decreased during the same period, with adjusted OR of 0.82 (95% CI = 0.78, 0.86) for each advanced study year.

**Table 2:** Odds Ratios (OR) of Type of Initial Concussion-related Visits, 2012–2017 (*n* = 4184)

	ED and Urgent Care vs. All Others			Sports Medicine Clinic vs. All Others		
	Adjusted OR^a^	(95% CI)	*p*-value	Adjusted OR^a^	(95% CI)	*p*-value
Insurance Type						
Private	0.74	(0.60, 0.90)	0.0002	1.45	(1.20, 1.76)	0.0002
Public	Ref			Ref		
Sex						
Male	Ref			Ref		
Female	0.97	(0.82, 1.14)	0.6959	1.00	(0.86, 1.18)	0.9711
Age, y						
10–13	Ref			Ref		
14–17	0.30	(0.25, 0.34)	< 0.0001	3.26	(2.81, 3.78)	< 0.0001
Race						
White	Ref			Ref		
Black	1.33	(1.03, 1.71)	0.0290	0.74	(0.58, 0.95)	0.0158
All others	1.15	(0.87, 1.52)	0.3344	0.88	(0.67, 1.16)	0.3604
Study Year^b^	1.19	(1.13, 1.25)	< 0.0001	0.82	(0.78, 0.86)	< 0.0001
Season						
Quarter 1	Ref			Ref		
Quarter 2	0.84	(0.65, 1.09)	0.1889	1.20	(0.94, 1.54)	0.1429
Quarter 3	0.91	(0.73, 1.13)	0.3973	1.09	(0.88, 1.34)	0.4295
Quarter 4	0.84	(0.67, 1.06)	0.1370	1.17	(0.94, 1.46)	0.1582

Note. *OR* Odds Ratio; *CI* Confidence Interval

a. Odds ratio was based on logistic regression adjusted for all the variables listed in the table

b. 2012 included data from Quarter 3 and 4 only

### Time from injury to initial concussion visit by insurance type

**Fig. 3:**
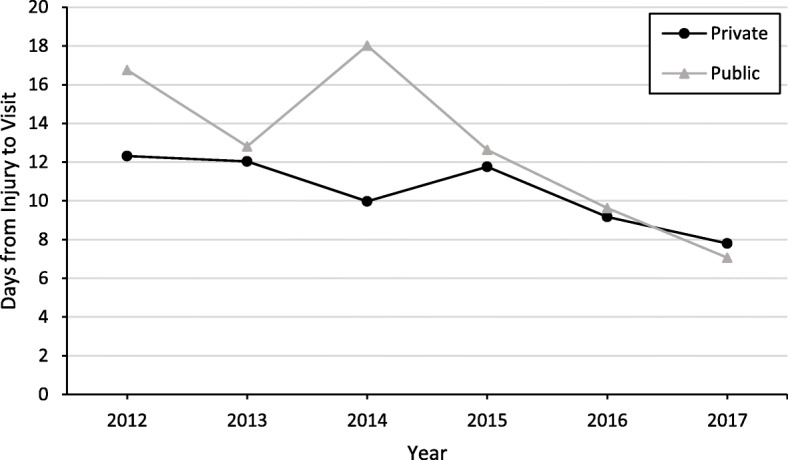
Days from Injury to Initial Concussion-related Visit, 2012–2017 (*N* = 4184)

The time from injury to initial concussion visit significantly decreased among both insurance groups throughout the study (β = − 0.1821, *P* < .0001) (Fig. 3). Furthermore, privately insured youth had a significantly shorter time from injury to initial visit than publicly insured youth (β = − 0.3842, *P* = .0008). Finally, a significant interaction between study year and insurance type was observed (β = 0.0807, *P* = .0106), with a significantly larger decrease in days from injury to initial concussion visit from 2012 to 2017 in publicly insured youth (16.8 to 7.1 days) than privately insured youth (12.3 to 7.8 days).

## Discussion

This study used a linked EHR system to evaluate the influence of insurance type on the rate, type, and time of initial medical visit following a concussion in a large pediatric hospital network between 2012 and 2017. The results of the study supported all three of our hypotheses. We found that privately insured youth had a significantly higher rate of initial concussion visits throughout the study than publicly insured youth. Furthermore, publicly insured youth were significantly more likely to seek initial concussion-related care at the ED and urgent care but less likely to seek initial concussion-related care at a sports medicine clinic than privately insured youth. Finally, days from injury to initial concussion visit significantly decreased among both insurance types throughout the study, with a greater decrease observed in publicly insured youth. Our study demonstrates that the rate, type, and time of initial concussion-related visits are different between insurance types in a large pediatric healthcare network. Our study leveraged the EHR of a large pediatric healthcare network to capture youth concussion care-seeking behaviors beyond the emergency setting, including the outpatient setting, where the majority of pediatric patients seek concussion care [1, 25].

The higher rate of initial concussion visits in the privately insured than the publicly insured youth observed in this study may be explained by greater participation in sports and more weekly hours in organized sports among youth of higher-income households [26]. Considering that injury during sports is the primary cause of concussion in youth and adolescents, privately insured youth would be more likely to sustain a concussion than publicly insured youth [16, 27, 28]. In studies of multiple states, enactment of concussion laws initially increased concussion-related visits, with the rate leveling-off or decreasing after a few years into the post law-period [9–11]. Though heightened public awareness as a result of educational efforts such as the Centers for Disease Control and Prevention’s “Heads Up” campaign [29] is cited as a reason for increased concussion-related visits, [10, 11, 15] the decreased rate observed in our study is consistent with prior studies of the Ohio law, which reported overall decreases in concussion-related visits in sports medicine clinics and EDs after the law’s passage [14, 15]. The observed decreased rate may be due in part to the guidelines on minimal body contact during sport practices [3] or the decrease in contact-sports participation across the nation [30, 31] which could be responsible for the greater decrease in rate of concussion visits seen in privately insured youth as well. Future studies should identify both the mechanism of injury and type of sport precipitating the concussion to better describe these evolving trends.

Consistent with previous study findings, [32] we found that publicly insured youth were more likely to seek initial concussion-related care at the ED and urgent care than privately insured youth. Previous studies show that publicly insured children are more reliant on the ED for medical care than those with private insurance [32] and that publicly insured pediatric patients increased ED visits relative to privately insured after the passage of concussion laws [22]. We also found that youth with public insurance were less likely than those with private insurance to visit a sports medicine clinic for their initial concussion-related care although the sports medicine clinic remained the main location for the majority of initial concussion visits throughout the study regardless of insurance type. A study of another pediatric healthcare network found primary care as the initial point of healthcare entry for 82% of a pediatric patients [23]. However, our study did not capture the patterns of primary care visits due to our inclusion criteria. Furthermore, the prior study reported a decrease in use of ED and urgent care as a point of healthcare entry from 2010 to 2014 for both publicly and privately insured [23]. The study’s inclusion criteria was limited to patients whose primary care was normally delivered by a pediatrician within the hospital network. Likewise, our study was limited to those who had at least one concussion-related medical visit at one of seven NCH sports medicine clinics. Thus, patients who sought initial care from their primary care provider may be less likely to be referred to sports medicine clinics if an uncomplicated concussion was experienced. Therefore, our study population is likely more symptomatic than youth with uncomplicated concussions managed by primary care physicians [33]. Because the concussion law requires medical clearance to return to play, both privately and publicly insured youth may seek care in the ED because it is perceived to expedite the processes of receiving medical clearance and the ability to return to play. Given ED visits incur high medical costs, the reliance of primary care as the dominant location for uncomplicated concussion care may be an easily accessible and low-cost approach [23, 24, 34, 35]. Primary care providers should be trained to evaluate and manage concussions during both the initial presentation and clinical follow-up [36].

The observed decrease in average time from injury to initial concussion visit for both insurance types is likely a consequence of the increase in initial concussion visits to the ED and urgent care. Emergency care is often sought in time-sensitive cases, and parity in time to initial visit is expected among public and private insurance types, regardless of ability to pay [37]. Though the time from concussion injury to initial visit for the publicly insured was longer than privately insured at the start of the study, the trends converged and crossed later in the study (Fig. 3). This change is likely due to the greater increase in initial concussion visits in the ED and urgent care among the publicly insured. A greater urgency for an initial visit may also be due to mandated medical clearance in concussion legislation and increased education for parents about concussion and its consequences [38, 39]. A shorter time to first visit following concussion is associated with faster recovery times in previous studies, so future studies should investigate how insurance type affects differences in care received until symptom resolution [14, 40]. Another possible explanation for the observed decrease in average time to initial concussion visit is an increase in availability of concussion care at NCH, although this study did not collect data on that aspect. Further investigation is merited to determine the change in access and availability of care on the time from injury to initial care visit.

It is important to note that both type of insurance and mechanism of injury may affect when and where initial concussion care is sought. Previous studies found that publicly insured youth are more likely than privately insured youth to have non-sports-related concussion [27, 41]. Thus, if publicly insured youth have concussions resulting from non-sports events, such as motor vehicle crashes, it is reasonable to assume that they would be more likely to seek initial medical care at an ED or urgent care rather than at a sports medicine clinic. Conversely, if privately insured youth suffer a sports-related concussion, they may be more likely to be referred by an athletic trainer to a sports medicine clinic for treatment. Additional research is needed to assess how insurance type may influence the location, type and time of concussion care while accounting for mechanism of injury.

Our study should be interpreted with several limitations. First, by using the EHR system of a single healthcare network, visits outside of the network were not included in our analysis. ICD-9 codes do not include the specificity of an initial medical encounter and ICD-10 codes define an initial medical encounter regardless of time of the injury. Therefore, using ICD codes to define concussion-related initial medical visit within the NCH network may not capture the true initial visit. Our inability to capture youth who had their initial concussion visits outside of the NCH network means that less symptomatic patients may have been cared for by providers who were not captured in our analysis, such as primary care. These exclusionary biases decrease the generalizability of our findings. Second, our study was retrospective which introduces concerns of accuracy in medical coding and detail. Third, we did not report the severity of concussion or the mechanism of the injury, both of which likely influence the choice of setting and time to initial concussion visit. Fourth, we limited our inclusion criteria to youth with at least one visit at an NCH sports medicine clinic, potentially tilting our population toward a cohort with a greater burden of symptoms. Fifth, we did not examine self-pay or mixed-insurance status as a category due to small cell size. Therefore, uninsured children were not evaluated, omitting representation of the millions of children who remain uninsured even when eligible for public insurance [42]. Finally, our study was limited to a pediatric hospital network in central Ohio that serves those in rural, suburban, and urban areas; as such it may not be generalizable to the greater pediatric population.

## Conclusions

This study evaluated initial concussion visits within a large pediatric network and used a unified EHR to determine differences in the care-seeking behaviors of publicly and privately insured youth. From 2012 to 2017, publicly insured youth had lower rates of initial concussion visits, were more likely to visit the ED and urgent care for initial concussion-related care and had a greater decrease in average time to initial concussion visit compared to privately insured youth. The differences observed between insurance types in this study along with general trends for both insurance types require further exploration. Specifically, future studies should include the data on mechanism of injury (i.e. sport-related vs. non-sports-related concussions) and should also capture a true initial medical visit following a concussion regardless of the in- or out-of- network status of the provider. Application of these findings may help improve the care of youth following concussion injury.

## Data Availability

The datasets generated and/or analysed during the current study contain clinical information and are not publicly available due to privacy or ethical restrictions but are available from the corresponding author on reasonable request.

## Abbreviations

CIs: Confidence intervals
ED: Emergency department
EHR: Electronic health records
ICD-9-CM: International classification of diseases, ninth revision
ICD-10-CM: International classification of diseases, tenth revision
NCH: Nationwide children’s hospital
OR: Odds ratio
